# Matrix Selection Strategies for MALDI-TOF MS/MS Characterization of Cyclic Tetrapyrroles in Blood and Food Samples

**DOI:** 10.3390/molecules29040868

**Published:** 2024-02-15

**Authors:** Mariachiara Bianco, Giovanni Ventura, Cosima Damiana Calvano, Ilario Losito, Tommaso R. I. Cataldi, Antonio Monopoli

**Affiliations:** 1 Department of Chemistry, University of Bari Aldo Moro, 70126 Bari, Italy; mariachiara.bianco@uniba.it (M.B.); giovanni.ventura@uniba.it (G.V.); ilario.losito@uniba.it (I.L.); tommaso.cataldi@uniba.it (T.R.I.C.); 2 Interdepartmental Research Center (SMART), University of Bari Aldo Moro, 70126 Bari, Italy

**Keywords:** phthalocyanine, heme, blood, food, MALDI MS/MS

## Abstract

Cyclic tetrapyrrole derivatives such as porphyrins, chlorins, corrins (compounds with a corrin core), and phthalocyanines are a family of molecules containing four pyrrole rings usually coordinating a metal ion (Mg, Cu, Fe, Zn, etc.). Here, we report the characterization of some representative cyclic tetrapyrrole derivatives by MALDI-ToF/ToF MS analyses, including heme b and c, phthalocyanines, and protoporphyrins after proper matrix selection. Both neutral and acidic matrices were evaluated to assess potential demetallation, adduct formation, and fragmentation. While chlorophylls exhibited magnesium demetallation in acidic matrices, cyclic tetrapyrroles with Fe, Zn, Co, Cu, or Ni remained steadfast against demetallation across all conditions. Phthalocyanines and protoporphyrins were also detectable without a matrix using laser desorption ionization (LDI); however, the incorporation of matrices achieved the highest ionization yield, enhanced sensitivity, and negligible fragmentation. Three standard proteins, i.e., myoglobin, hemoglobin, and cytochrome c, were analyzed either intact or enzymatically digested, yielding heme b and heme c ions along with accompanying peptides. Furthermore, we successfully detected and characterized heme b in real samples, including blood, bovine and cod liver, and mussel. As a result, MALDI MS/MS emerged as a powerful tool for straightforward cyclic tetrapyrrole identification, even in highly complex samples. Our work paves the way for a more comprehensive understanding of cyclic tetrapyrroles in biological and industrial settings, including the geochemical field, as these compounds are a source of significant geological and geochemical information in sediments and crude oils.

## 1. Introduction

**Figure 1:**
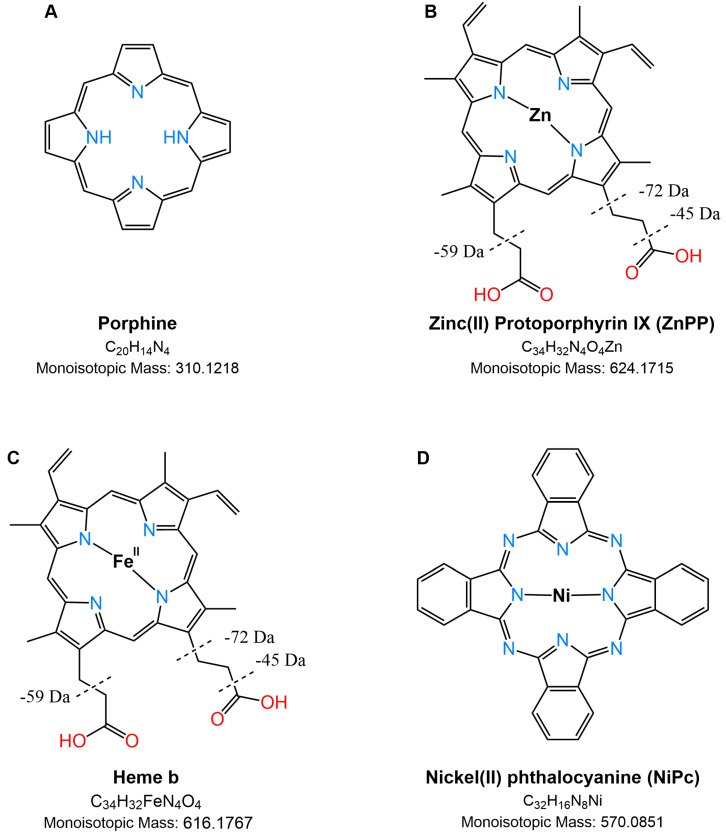
Structures of some compounds investigated with porphine core (**A**), such as Zinc(II) protoporphyrin IX (ZnPP) (**B**), heme b (**C**), and nickel(II) phthalocyanine (**D**). The major neutral losses of ZnPP and heme b are reported as dashed lines; note that some ions could be generated from the concurrent loss of reported fragments (see Table 1).

Porphyrins are a group of macromolecules participating in the biosynthesis of numerous fundamental biomolecules, such as vitamin B_12_ [1] and heme [2]. At the heart of all porphyrins lies a central tetrapyrrolic ring, *porphine* (Figure 1A), held together by methane bridges. The unique identity of each porphyrin species stems from its side chain substituents, which can be alkyl, alkene, or carboxylic acid groups. As an example, protoporphyrin IX (Figure 1B) [3] is an organic compound classified as a porphyrin containing four methyl (M), two vinyl (V), and two propionic acidic (P) groups; it plays an important role in living organisms as a precursor of heme b (Figure 1C) and chlorophyll. Many metal ions can be incorporated into porphyrins generating metalloporphyrins, whose stability can vary dramatically. For instance, Mg(II) porphyrins are easily demetallated by weak acidic conditions, being the most labile metalloporphyrins [4]. Naturally occurring porphyrinic molecules constitute the most important class of biological cofactors present as green pigments in chloroplasts [5] or red pigments in hemoglobin (heme b), myoglobin (heme b), and cytochrome c (heme c) [6,7]. Heme, the iron-containing prosthetic group of many proteins, including hemoglobin, is essential for oxygen transport and other crucial biological processes. However, the redox activity of iron and the hydrophobic nature of protoporphyrin IX can lead to cytotoxicity effects, which can be minimized when heme is bound to proteins. Therefore, analyzing unbound heme levels in patient plasma samples can provide valuable insights into conditions like hemolysis, where heme is released from its bound state [7,8,9].

Synthetic porphyrins and aromatic tetrapyrrole (i.e., corrole) complexes have been proposed for diverse applications, serving as biomimetic models, catalysts for many reactions [10], sensors [11], and therapeutic agents [12]. Phthalocyanines (Figure 1D), which are closely related to porphyrins, have rich synthetic chemistry and have been widely used as dyes for textiles and paper, as blue and green colors in liquid crystal displays, as photoconductors in laser printers, and as absorbers and *p*-conductors in organic solar cells [13]. Such an outstanding collection of properties allows porphyrin derivatives to be applied to different transducers, ranging from nano-gravimetric to optical devices, and empowering the conception of multiple transduction chemical sensors onto the same sensing layer. The ability to modify the structure of the macrocycle by synthetic alteration of the various components of the porphyrin ring, including the molecular skeleton, substituents, and coordinated metals/metalloids in the ligand, offers a vast range of possibilities for creating porphyrinoid-based systems with precisely tailored properties. This provides the opportunity to generate a massive library of such systems with finely tuned properties [14].

Optical spectroscopic techniques such as UV–vis spectrophotometry, chemiluminescence, and fluorescence are widely preferred for the identification of these porphyrinic compounds, due to their extensive system of delocalized π electrons and fluorescent nature [15,16]. However, these currently used techniques may not guarantee a high level of selectivity. This is because they do not provide detailed information on the molecular structure of the analyzed compounds. This can be a limiting factor when examining newly synthesized derivatives. Additionally, in complex natural samples, interference from other pigments and degradation by-products may occur. A more selective method for the separation of cyclic tetrapyrroles and related compounds is reversed-phase high-performance liquid chromatography [17] coupled with mass spectrometry (RP-HPLC-MS) [18,19], even if, due to the time-consuming sample treatment and experimental setup, routine analyses of a significant number of samples become unaffordable. In this context, matrix-assisted laser desorption/ionization (MALDI) time-of-flight (ToF) MS can offer a fast and selective technique for the identification of cyclic tetrapyrroles thanks to well-recognized features, such as speedy and simple sample preparation, tolerance to salts, and high sensitivity [20]. Indeed, recent works employing MALDI-MS for the analysis of cyclic tetrapyrrole derivatives have been published [8,21,22,23,24,25,26]. We have also deeply demonstrated that a major challenge in the MS analysis of porphyrinic compounds is to preserve the original metallation state during the analysis, avoiding demetallation or trans-metallation of metalloporphyrins and metallation of free-base porphyrins [27,28,29]. Reflecting the extensive conjugation of these systems and the nature of the coordinating metal cation, numerous ionization pathways can occur during the MALDI process. Thus, the properties of the MALDI matrix (proton or electron transfer) can be crucial to their successful ionization [30].

To guarantee a high degree of selectivity through molecular mass, isotopic distribution, and structure-specific fragmentation, accurate tandem mass spectrometry is needed, but detailed structural studies by MALDI MS/MS investigation are limited. Here, we report the examination and comparison of various recognized MALDI matrices for the identification and characterization of several porphine-derived compounds, also employing tandem MS analysis. The results demonstrate that MALDI MS/MS also allowed the complete, rapid, and incisive description of these compounds in complex food and biological samples after selecting the best matrix for each group.

## 2. Results and Discussion

### 2.1. Matrix Evaluation for Protoporphyrin Analysis by MALDI MS

**Figure 2:**
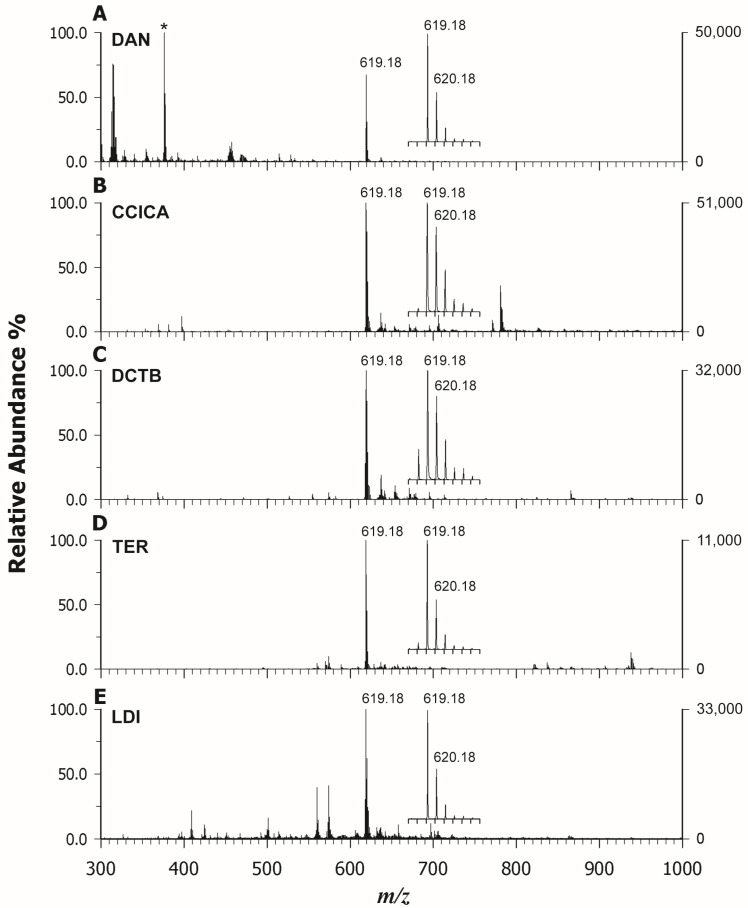
Positive ion mass spectra by MALDI-ToF MS of a *CoPP* using (**A**) DAN, (**B**) CClCA, (**C**) DCTB, and (**D**) TER as matrices. LDI mode is reported in (**E**). The expanded isotopic pattern of the odd-electron [M]^+•^ radical at *m*/*z* 619.18 is shown in the inset, (*) main peak.

In the first step, the desorption/ionization efficiency of representative matrices for analyzing a synthetic Co(II) protoporphyrin was evaluated and the results obtained with and without a matrix were compared. Alongside conventional low-proton-affinity (PA) acidic matrices such as α-cyano-4-hydroxycinnamic acid (CHCA) and α-cyano-4-chlorocinnamic acid (CClCA), known to facilitate magnesium porphyrin demetallation [28], neutral or electron transfer (ET) matrices like DCTB (trans-2-[3-(4-t-butyl-phenyl)-2-methyl-2-propenylidene]malononitrile), DAN (1,5-diaminonapthalene), and TER (2,2′:5′,2″-terthiophene) were tested to ensure the stability of the central metal ion and to identify potential matrix-related interfering peaks in the low *m*/*z* range (<1000 *m*/*z*). Figure 2 displays the MALDI MS spectra of CoPP (cobalt chloride (II) Protoporphyrin IX) in positive ion mode, using DAN (A), CClCA (B), DCTB (C), and TER (D) as matrices, or in LDI mode (E). In all cases, CoPP was detected as the base peak at *m*/*z* 619.18, except for the MALDI spectrum obtained using DAN as the matrix (Figure 2A), where the main peak (*) is generated from an interfering matrix ion. From the MALDI mass spectra, it is clear that Co-porphyrin is relatively stable and a demetallation reaction does not occur when also employing acidic matrices. Indeed, Buchler et al. [31] developed a stability index (S) to evaluate the stability of metal–porphyrin complexes, which is related to certain metal properties, i.e., Pauling electronegativity (PE), valence (V), and ionic radius expressed in Å (R). The index S can be calculated as S = ((PE × V)/R). The higher the stability index, the lower the possibility of demetallation in the solution. Considering the relevant values for Mg(II) (R = 0.72 Å, PE = 1.31, V = 2), Co(II) (R = 0.65 Å, PE = 1.88, V = 2), and Zn(II) (R = 0.74 Å, PE = 1.65, V = 2), the porphyrin cycles containing Co and Zn as the central metals are more stable than the one containing Mg, with the stability index, S being, respectively, 5.78 (Co) > 4.46 (Zn) > 3.64 (Mg).

**Figure 3:**
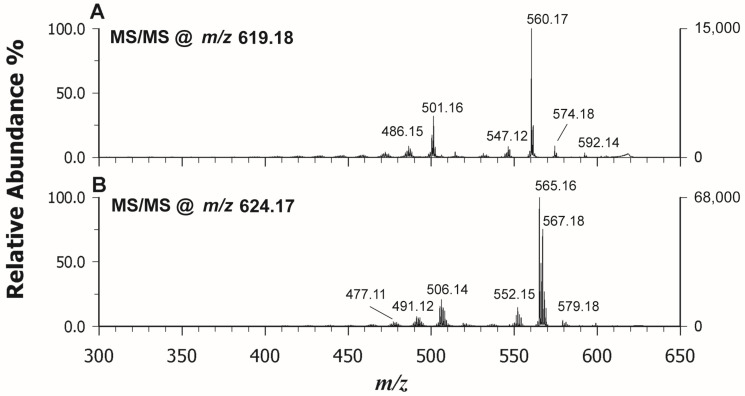
MALDI-ToF/ToF tandem mass spectra in positive ion mode of *CoPP* at *m*/*z* 619.18 (**A**) and ZnPP at *m*/*z* 624.17 (**B**). CClCA was used as a matrix; peaks are indicated according to their *m*/*z* value and their identities are given in Table 1.

As evidenced in the insets of Figure 2, alongside the M^+•^ at *m*/*z* 619.18, the M + 1 isotope exhibited a relative abundance of ca 85% in Figure 2B,C, which is higher than the expected natural isotopic abundance of 36.8%, thus indicating the major contribution of a simultaneously formed protonated molecule ([M + H]^+^). Thus, while DAN (A) and TER (D) could drive the ionization process towards the formation of the odd electron molecular ion with nearly 100% yield as in LDI mode (E), using CClCA (B) and DCTB (C), a concurrent ionization with the formation of a protonated adduct was observed. Proton transfer (PT) ions tend to be energetically preferred for the classical matrices CClCA and CHCA, which have acidic sites, while ET ions can be dominant for other matrices (DCTB, DAN, TER), and both types may appear in the same spectrum. The propensity for gas-phase PT or ET depends on the relative ionization energies, electron and proton affinities, and basicity of the interacting partners, even if the interconversion reactions are likely for many matrices in the early plume being rather low in energy [32]. In our case, ET is the favored mechanism considering the highly aromatic feature of all cyclic tetrapyrroles, but it competes with PT when CClCA and DCTB are used as matrices likely due to the presence of two carboxylic end residues in the PPIX structure (see Figure 1B). The intensity of the CoPP is highest when analyzed with CClCA (B) and is comparable to LDI (C) when using DAN (A) and DCTB (C), even if fewer matrix-related peaks or fragment ions occurred using the latter matrix instead of DAN and LDI, respectively. TER (D) gave rise to the lowest signal in terms of intensity. It should be noted an increased contribution from the peak at *m*/*z* 618.17 that corresponds to [M − H]^+^ for DCTB, suggesting that also a PT from analyte to matrix can compete with the ET process. Indeed, the loss of a hydrogen radical (H^•^) from the retinol molecular radical cation was already reported by Cole et al. [33] and by O’Connor et al. [34] in chlorophyll-a, facilitated by the generation of thermodynamically favored conjugated structures. To avoid this further complication, CClCA exhibited the best performance as the PT matrix and DAN as the ET matrix for *CoPP*. Overlapping results were obtained for the other investigated standard of Zn(II) Protoporphyrin IX (ZnPP), thus confirming our findings. Then, we tested these matrices for tandem MS acquisition to verify if useful spectra for structural information could be attained. All the matrices were able to generate very similar fragmentation patterns, even if CClCA produced spectra with higher intensity, so we referred to this matrix. Figure 3 reports the MS/MS spectrum of CoPP (A) and ZnPP (B) in positive ion mode. The fragmentation pattern of the porphyrins is dominated by cleavages of the side chain substituents, so the investigation of the product ion MALDI spectrum allows the identification of the substituent groups. In analogy with ESI-MS/MS [17], an acetic acid substituent typically eliminates a neutral H_2_CO_2_ (46 Da) while a propionic acid substituent loses preferentially a H_3_C_2_O_2_ radical ([CH_2_COOH]^•^ 59 Da) group. Porphyrins containing both acetic and propionic acid substituents show a combination of both pathways. The same findings apply to both ZnPP and CoPP, and the main product ions identified in Figure 3 are listed in Table 1.

### 2.2. MALDI MS(/MS) of Phthalocyanines

**Figure 4:**
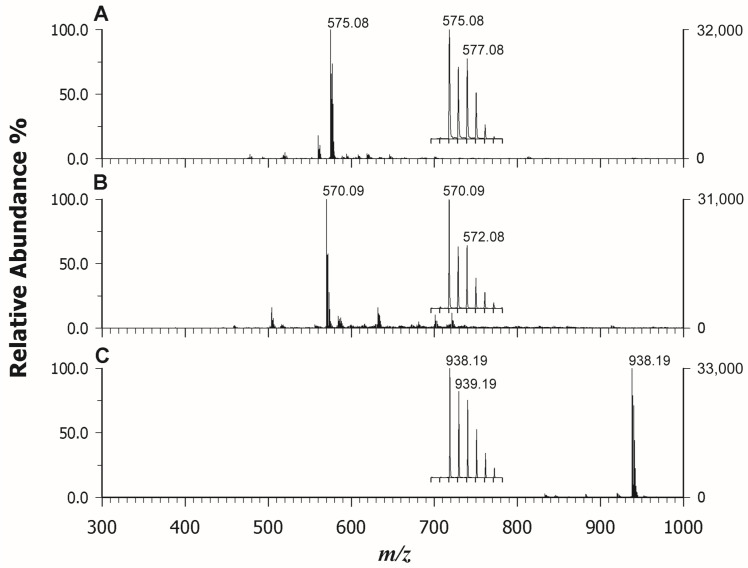
LDI-ToF mass spectra in positive ion mode of *CuPc* (**A**), *NiPc* (**B**), and *NiPcPhe* (**C**) with corresponding insets on the base peak.

**Figure 5:**
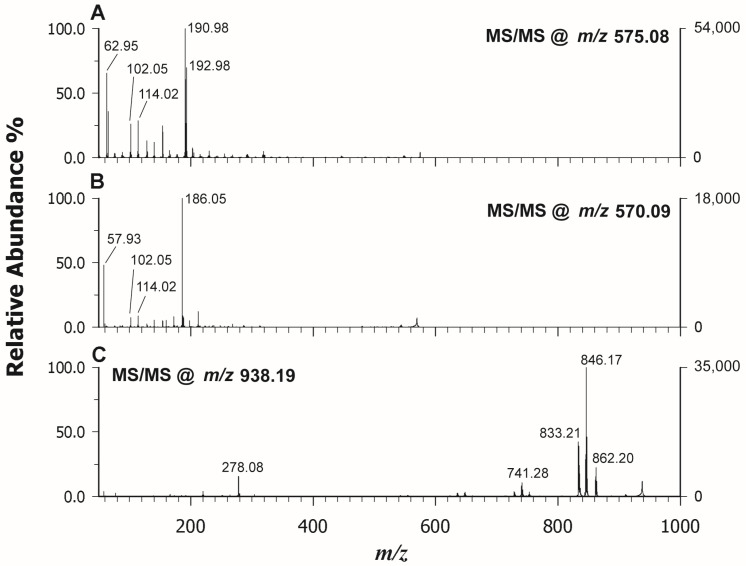
LDI-ToF/ToF mass spectra in positive ion mode of *CuPc* at *m*/*z* 575.08 (**A**), *NiPc* at *m*/*z* 570.09 (**B**), and *NiPcPhe* at *m*/*z* 938.19 (**C**).

Phthalocyanines (Pcs) are largely employed in several applications in modern technology from sensors [11] to cultural heritage in street art [35] for their interesting spectroscopic, optical, and electrical properties in addition to chemical and thermal stability. Also in this case, the MALDI MS analysis offers the advantage of a fast analysis with high sensitivity that can provide structural information on chemical species and relative quantification information during the synthesis of novel Pcs with improved performance. In a previous study [36], metal phthalocyanines (MPcs) have been used as a matrix for the analysis of small molecules (<500 Da) able to form spotless peaks free of interference of MPc–analyte adducts in a mass region of >1000 Da. Due to the highly conjugated π system, MPcs show a Soret absorption band between 290 nm and 450 nm, and thus they can easily absorb laser energy. For this reason, we should expect that PCs can be detected well also in the LDI modality. Indeed, after exploring all the ET or PT matrices listed in the Section 3, we confirmed that the three investigated PCs with different central metal ions (Cu vs. Ni) or different lateral substituents were significantly ionized in LDI mode with high intensity and without matrix ion interference. Figure 4 reports the LDI spectra in positive mode of Copper (II) phthalocyanine (CuPc) (A), Nickel(II) phthalocyanine (NiPc) (B), and Nickel(II) 2,9,16,23-tetraphenoxy-29H-31H-phthalocyanine (NiPcPhe) (C). In all cases, the base peak detected for each compound was represented by the radical cation at *m*/*z* 575.08 (CuPc), 570.09 (NiPc), and 938.19 (NiPcPhe), reflecting the natural isotopic pattern. The use of matrices did not add any improvement, since the occurrence of interfering ions was experienced together with the concurrent formation of protonated adduct that complicated the spectrum. Moreover, the Pcs have scarce solubility in the common organic solvents used to dissolve matrices, so, in some cases, on-target precipitation reactions were also surveyed. The MS/MS analyses of the selected compounds were also carried out in LDI mode and Figure 5 reports the relevant spectra of CuPc (A), NiPc (B), and NiPcPhe (C). The tandem mass spectra did not provide useful information on the structure since Pcs have great thermal stability and usually spectral studies on metal phthalocyanine are established to be meagre. However, our findings were in agreement with the NIST library, where very similar electron ionization spectra of CuPc [37] and NiPc [38] are found. Some tentative fragmentation mechanisms and attributions were proposed by Achar et al. [39] reporting GC–MS studies of nickel phthalocyanine sheet polymer. The MALDI spectrum of the substituted NiPcPhe looks different, since fragments linked to the consecutive losses of phenyl and phenoxyl alongside CO residues were detected in the *m*/*z* range 600–863 (Figure 5C). In this case, MALDI MS/MS can be exploited to obtain more information on the nature of substituents.

### 2.3. MALDI MS(/MS) of Heme b and c in Standards and Real Samples

**Figure 6:**
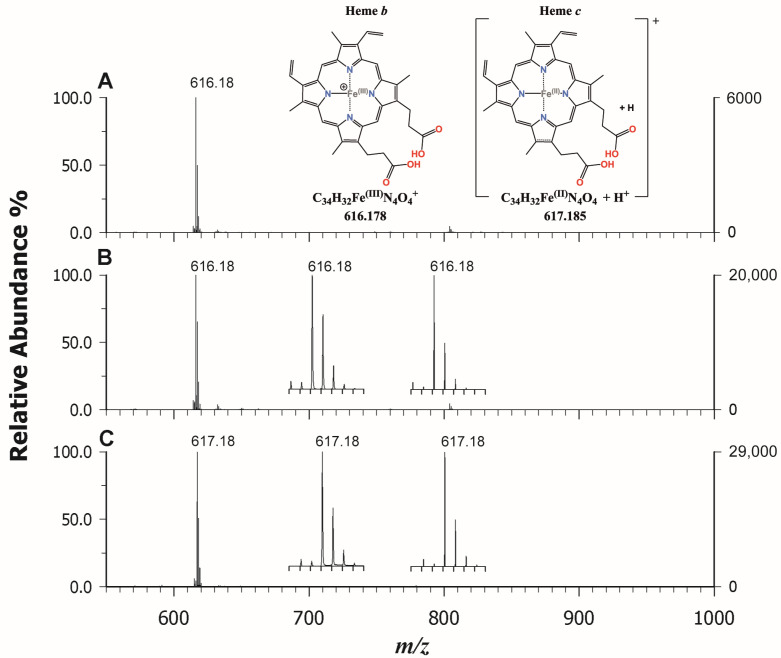
MALDI-ToF mass spectra in positive ion mode using CHCA as a matrix of heme b in hemoglobin (**A**), in myoglobin (**B**), and heme c in cytochrome C (**C**). The comparison between experimental and theoretical mass spectra is shown in the insets of plots (**B**) and (**C**).

A complete characterization of the heme molecule is important, as it can provide a rapid method of disease detection, structural information on unusual heme groups, evidence on association of drugs with heme groups, or data on hemoproteins with specific biological functions [40,41]. Myoglobin and hemoglobin were here used as reference materials containing the heme b moiety, while cytochrome C (Cyt C) was chosen as a reference for heme c. After screening ET and PT matrices (see Figures S1 and S2), we noticed that heme was successfully and efficiently detected by employing PT matrices such as CClCA and CHCA. Furthermore, the use of this last matrix allowed us to systematically obtain suppression of other interfering species in support of heme ionization. In Figure 6, the MALDI MS spectra in the low *m*/*z* regions of hemoglobin (A), myoglobin (B), and cytochrome c (C) using CHCA as a matrix are reported. In detail, the intact analyte heme b (Figure 6A,B) was identified at *m*/*z* 616.18, which can be rationalized by admitting the occurrence of an Fe^3+^ ion within the intact porphyrin cycle, such as [C_34_H_32_O_4_FeN_4_]^+^. Indeed, it is known that the Fe^2+^ within the cycle easily oxidizes in the air or during the analyses [42]. A comparison between the theoretical intensity of the second isotopic peak of heme b (40% of the peak at *m*/*z* 616) and that obtained experimentally (ca 60–70%) clearly shows, in both cases, the presence of an unknown ionic species formed at *m*/*z* 617.18. This phenomenon can be partially explained as the overlapping problem of isobaric species which causes a change in the apparent isotopic distribution, as proved by Yang et al. [24] comparing the high-resolution mode (~800,000) in MALDI FT-ICR-MS and low-resolution mode (∼8000).

**Figure 7:**
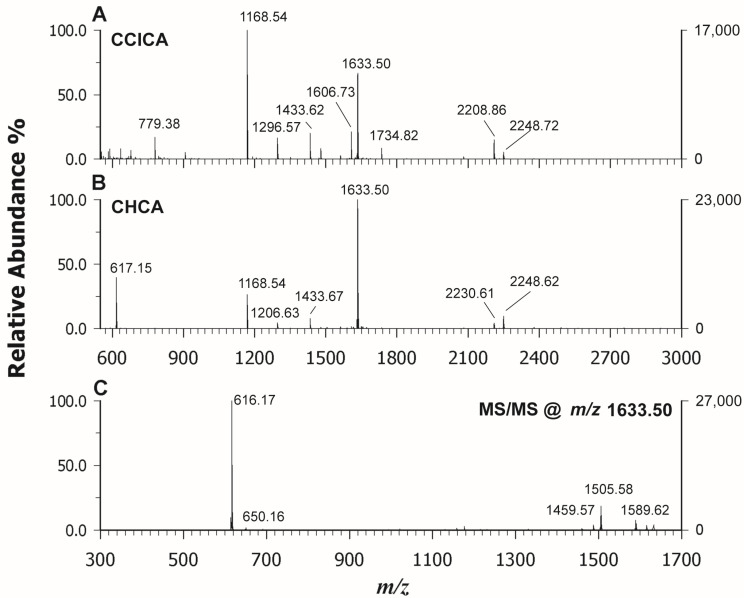
MALDI-ToF mass spectra in positive ion mode of a tryptic digest of cytochrome C analyzed using CClCA (**A**) and CHCA (**B**) as a matrix. The tandem MS spectrum of heme-modified peptide detected at *m*/*z* 1633.50 using CHCA as a matrix is shown in plot (**C**).

Upon examining Figure 6C, it is evident that the signal at *m*/*z* 617.18 represents the base peak in the dissociation of heme from cytochrome c. This signal poses a challenging explanation, as it requires the rupture of two covalent and two coordination bonds to release the porphyrin complex heme c. In the literature, it is reported that this species can be generated in electrospray (ESI) by collisionally induced dissociation (CID) following the ionization of intact cytochrome c [42]. In the MALDI process, the iron reduction within the complex may be due either to the relatively high electron affinity of the metalloporphyrins or to the presence of free electrons in the plume; however, an excess of energy is required to simulate CID conditions. When analyzing cytochrome C at low concentrations (<0.1 mg/mL) using our experimental setup, no signal attributable to heme c was detected until higher concentrations (1.0 mg/mL) were investigated. These findings suggest that the greater presence of neutral species within the MALDI plume, due to the concentration increase of the protein by a factor of 10, together with the higher laser energy used compared to hemoglobin and myoglobin, can permit the breaking of the covalent bonds releasing heme c. According to Crestoni et al. [43], the protonation of heme occurs at the level of one of the vinyl groups. We were intrigued by this odd behavior of heme c, and therefore we also investigated the complex bound to protein after its digestion by trypsin, following a classical bottom-up approach for peptide mass fingerprinting (PMF). The proteolysis was carried out on hemoglobin, myoglobin, and cytochrome c, and both CClCA and CHCA were employed as matrices. Also in this case, the use of CHCA allowed us to ionize the heme group very well alongside the peptides arising from each protein, which were still detectable but with less intensity. As an example, Figure 7 reports the PMF of Cyt C analyzed by using CClCA (A) and CHCA (B). It is possible to observe the occurrence of heme c at *m*/*z* 617.18 at a higher intensity in Figure 7B compared to Figure 7A where CClCA was employed, also confirming the efficacy of CHCA in ionizing the heme group in the complex sample. Further, it is interesting to note the base peak in Figure 7B at *m*/*z* 1633.50 which corresponds to a peptide ambiguously assigned to the sequence IFVQKCAQCHTVEK or CAQCHTVEK combined with a heme group [44]. To confirm the attribution, M/MS was performed, and the results are reported in Figure 7C. The manual inspection of the spectrum identifies the origin of the peak as CAQCHTVEK + heme, since some y ions bear the heme moiety [44] which was correspondingly observed as an intense fragment ion at *m*/*z* 616.17. Very fascinatingly, the energy applied for the dissociation of peptides is likely similar to the energy required to simulate CID conditions in ESI, and indeed the ion was detected as in hemoglobin and myoglobin [42]. The main difference between CHCA and CClCA relies on the different ionization yields of heme and heme-modified peptides. The coverage of PMF was higher using CClCA (88% vs. 66 % for cyt C), but the detection of iron-containing peptides was greatly enhanced without any further time-consuming enrichment procedure [45].

**Figure 8:**
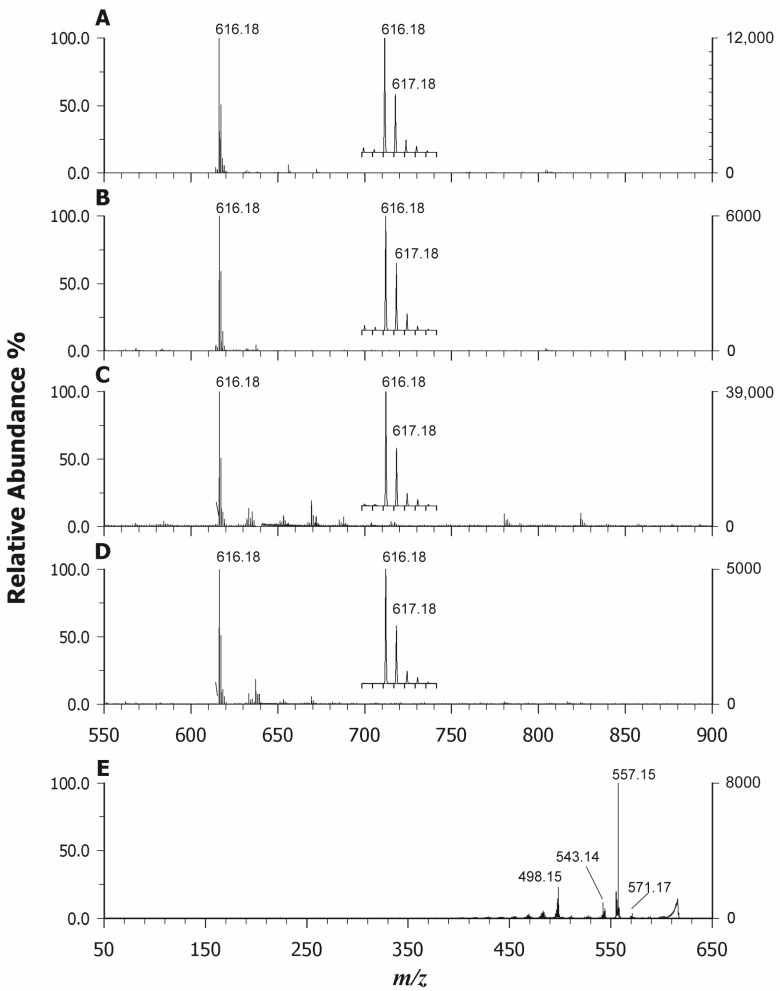
MALDI-ToF mass spectra in positive ion mode of blood (**A**), bovine liver (**B**), fish liver (**C**), and mussel (**D**) using CHCA as a matrix. (**E**) Tandem MS spectrum of heme b at *m*/*z* 616.18 using CHCA as a matrix.

Given the excellent properties of CHCA in the ionizing heme group, it was chosen for its analysis and MS(/MS) characterization on real samples. Figure 8 shows the MALDI MS spectra of blood (A), bovine liver (B), fish liver (C), and mussel (D) simply diluted in water as described in the Section 3. In all cases, the ion at *m*/*z* 616.18 was the base peak, suggesting the occurrence of heme b as an unbounded group likely in hemoglobin and myoglobin (in the liver). To confirm the attribution of heme, MS/MS data were collected for each sample and Figure 8E reports the spectrum of heme b in blood analyzed using an isolation window ±3 *m*/*z* units wide. The fragmentation spectrum obtained is identical to that obtained for heme b extracted from hemoglobin standard protein (see Figure S3). As can be seen, the base peak is represented by the ion fragment at *m*/*z* 557.15, due to a β-cleavage from one of the two carboxyl substituents with loss of a carboxymethyl radical and formation of the porphyrin radical cation stabilized by a π conjugated system which allows the delocalization of the unpaired electron. Another important peak is represented by the ion at *m*/*z* 543.14, a radical cation formed following the homolytic breaking of the bond between the porphyrin macrocycle and one propionic group. All other fragments, resulting from homolytic bond breaks, are explained in the inset of Figure S3. Some signals, however, are more difficult to interpret as the peak at *m*/*z* 498.15, for example, resulting from the simultaneous loss of both propionic groups, should be at *m*/*z* 499.15. One possible explanation may be the choice of the large isolation window which includes both *m*/*z* 616 and 617, as we also perceived the fragment at *m*/*z* 498.15 in the MS/MS spectrum of heme c.

## 3. Materials and Methods

### 3.1. Chemicals

Water, acetone, acetonitrile, trifluoroacetic acid (TFA), 2,2′:5′,2″-terthiophene (TER), 1,5-diaminonapthalene (DAN), trans-2-[3-(4-t-butyl-phenyl)-2-methyl-2-propenylidene]malononitrile (DCTB), 4-hydroxy-α-cyano cinnamic acid (CHCA), 4-chloro-α-cyano cinnamic acid (CClCA), Nickel(II) phthalocyanine (NiPc), Nickel(II) 2,9,16,23-tetraphenoxy-29H-31H-phthalocyanine (NiPcPhe), Cu(II) phthalocyanine (CuPc), Zinc(II) Protoporphyrin IX (ZnPP), Cobalt chloride (II) Protoporphyrin IX (CoPP), cytochrome c (horse heart), myoglobin, human hemoglobin, and proteomic-grade trypsin, were obtained from Sigma-Aldrich (Milan, Italy). Livers of cod and bovine as well as mussel were purchased from a local supermarket. Plasma was drawn from a healthy voluntary subject. All solvents used were LC–MS grade. A mass standards kit containing bradykinin (2–9 clip), angiotensin I, glu1-fibrinopeptide, ACTH (1–17, 18–39, 7–38 clips) for calibration was purchased from AB Sciex (Concord, ON, Canada).

### 3.2. Instrumentation

All experiments were performed using a 5800 MALDI ToF/ToF analyzer (AB SCIEX, Darmstadt, Germany) equipped with a neodymium-doped yttrium lithium fluoride (Nd:YLF) laser (345 nm), in reflectron positive mode with a typical mass accuracy of 5 ppm. In MS and MS/MS mode, 1000 laser shots were normally accumulated by a random rastering pattern at laser pulse rates of 400 and 1000 Hz, respectively; the mass spectra shown in this paper were averaged on at least five single mass spectra (1000 laser shots each). MS/MS experiments were performed setting a potential difference of 1 kV between the source and the collision cell; collision-induced dissociation (CID) modality was activated using argon as the collision gas with a medium pressure of 10^−6^ Torr. The delayed extraction (DE) time was set at 400 ns. The laser fluences used were fixed close to the laser threshold for each matrix within a range of 1.9–2.5 J/m^2^. DataExplorer software 4.0 (AB Sciex) was used to control the acquisitions and to perform the initial elaboration of data, while SigmaPlot 14.0 was used to graph the final mass spectra. ChemDraw Pro 8.0.3 (CambridgeSoft Corporation, Cambridge, MA, USA) was employed to draw chemical structures.

### 3.3. Sample Preparation

#### 3.3.1. Standard Phthalocyanines and Protoporphyrins

Nickel(II) phthalocyanine, Nickel(II) 2,9,16,23-tetraphenoxy-29H-31H-phthalocyanine, Copper(II) phthalocyanine, and Zinc(II) protoporphyrin IX were prepared at 1 mg/mL concentration in THF. For cobalt chloride (II) protoporphyrin IX, a 0.5 mg/mL solution was prepared since it tended to precipitate at higher concentrations. For MALDI analyses, 5 μL of the resulting solutions were mixed with an equal volume of each matrix solution (DAN, DCTB, TER, CHCA, and CClCA, 5 mg/mL in acetone); 1 μL of this solution was then spotted onto a MALDI plate and allowed to dry. Analyses also in LDI modality were carried out by depositing 1 μL of each standard solution directly on the target.

#### 3.3.2. Standard Hemoglobin, Myoglobin, and Cytochrome c

Human hemoglobin, bovine myoglobin, and cytochrome c were prepared at a concentration of 0.5 mg/mL in water. For tryptic digestion, 50 μL of each solution was added with 2.5 μL of trypsin 0.1 g/L and incubated overnight in a thermo-shaker at 37 °C under stirring at 400 rpm. Alternatively, these solutions were subjected to microwave digestion at 37 °C for 1 h (800 W output at 60 Hz, AC 220–240 V) but digestion yield was not reproducible so only the samples digested overnight are discussed here. For MALDI analyses, 5 μL of the resulting solution (before and after digestion) were mixed with an equal volume of each matrix solution (DAN, CHCA, CClCA, DCTB, 10 mg/mL in 70:30 ACN:0.1% TFA); 1 μL of this solution was then spotted on to a MALDI plate and allowed to dry. Unless otherwise specified, the dried-droplet method was used throughout this work. Analyses in LDI modality were also carried out. WARNING: Caution should be taken when handling DAN due to possible carcinogenetic effects. Exposure to the chemical should be minimized and fume hoods should always be used.

#### 3.3.3. Heme in Blood and Food Samples

Blood samples were diluted 50 times with water and analyzed as such. Bovine and cod liver and mussel were homogenized in a mortar, and then 5 mg of each were dissolved in 1 mL of water. After centrifugation (4300× *g* for 10 min) the supernatant was collected and analyzed as such. All samples were also digested; 50 μL of each solution was added to 2.5 μL of trypsin 0.1 g/L and incubated overnight at 37 °C under stirring at 400 rpm. For MALDI analyses, 5 μL of the resulting solution (before and after digestion) were mixed with an equal volume of each matrix solution (CHCA and CClCA, 10 mg/mL in 70:30 ACN:0.1% TFA); 1 μL of this solution was then spotted on to a MALDI plate and allowed to dry.

## 4. Conclusions

In this study, we conducted a characterization of porphine-derivative compounds using MALDI MS/MS in positive ion mode, following a careful selection of the appropriate matrix. Our findings reveal that ET or PT matrices can be employed for protoporphyrin analysis, but for enhanced signals in both MS and MS/MS acquisitions, CClCA proves to be more effective. In the case of phthalocyanines, the LDI-MS is consistently preferable, mitigating interference from matrix-related peaks. This is crucial as phthalocyanines absorb laser energy significantly, allowing desorption and adequate internal energy for successive fragmentation. Despite the inherent stability of these compounds, achieving robust fragmentation spectra becomes challenging unless substituted phthalocyanine variants are explored. For the ionization of heme b and c, CHCA stands out as a MALDI matrix, demonstrating great utility in the analysis of iron-containing peptides. Remarkably, CHCA facilitates the easy detection of heme b even in complex samples such as blood and food, eliminating the need for time-consuming pre-treatment. By simply diluting the sample and applying CHCA as a matrix, we achieved reliable results. As we continue to explore the vast realm of porphyrin-like metabolites in nature or newly synthesized compounds, our study underscores the potency of MALDI-MS/MS when coupled with the right matrix. This combination proves to be a robust and sensitive technique for the successful characterization of these compounds in biological samples.

## Figures and Tables

**Table 1:** Product ions summary of Co(II) and Zn(II) protoporphyrins, both in positive ion mode, obtained by MALDI MS/MS using CClCA as a matrix (see Figure 3).

Product Ions	Proposed Formula	Loss of Chemical Species	Theoretical *m*/*z* Value	MALDI MS/MS	
				*m*/*z*	Relative Intensity%
CoPP ([C_34_H_32_CoN_4_O_4_]^+^) at *m*/*z* 619.180					
B	[C_32_H_29_CoN_4_O_4_]^+•^	[CH_2_CH]^•^	592.156	592.14	5.2
C	[C_33_H_31_CoN_4_O_2_]^+•^	[HCO_2_]^•^	574.177	574.18	9.2
D	[C_32_H_29_CoN_4_O_2_]^+•^	[CH_2_COOH]^•^	560.162	560.17	100
E	[C_31_H_28_CoN_4_O_2_]^+^	[CH_2_CHCOOH]	547.154	547.12	6.4
F	[C_30_H_26_CoN_4_]^+^	2×[CH_2_COOH]^•^	501.148	501.16	32.3
G	[C_29_H_23_CoN_4_]^+•^	[C_5_H_9_O_4_]^•^	486.125	486.15	8.7
ZnPP ([C_34_H_32_ZnN_4_O_4_]^+•^) at *m*/*z* 624.172					
B	[C_33_H_31_ZnN_4_O_2_]^+^	[HCO_2_]^•^	579.174	579.18	2.3
C	[C_32_H_29_ZnN_4_O_2_]^+^	[CH_2_COOH]^•^	565.158	565.16	100
D	[C_31_H_28_ZnN_4_O_2_]^+^	[CH_2_CHCOOH]	552.150	552.15	14.6
E	[C_30_H_26_ZnN_4_]^+•^	2×[CH_2_COOH]^•^	506.145	506.14	20.7
F	[C_29_H_23_ZnN_4_]^+^	[C_5_H_9_O_4_]^•^	491.121	491.12	7.6
G	[C_28_H_21_ZnN_4_]^+^	[C_6_H_11_O_4_]^•^	477.106	477.11	3.6

## Data Availability

The authors confirm that most of the data supporting the findings of this study are available within the article and its supplementary material. Raw data are available from the corresponding authors (C.D.C. and A.M.) on request.

## Supplementary Materials

The following supporting information can be downloaded at: https://www.mdpi.com/article/10.3390/molecules29040868/s1. Figure S1. MALDI-ToF mass spectra in positive ion mode using 2-[(2E)-3-(4-tert-butylphenyl)-2-methylprop-2-enylidene]malononitrile (DCTB) as a matrix of heme b in hemoglobin (A), in myoglobin (B), and heme c in cytochrome C (C). Figure S2. MALDI-ToF mass spectra in positive ion mode using CClCA as a matrix of heme b in hemoglobin (A), in myoglobin (B), and heme c in cytochrome C (C). Figure S3. Tandem MS spectrum of heme b from hemoglobin standard protein at *m*/*z* 616.18 using CHCA as a matrix.

## References

[1] Watanabe F., Yabuta Y., Bito T., Teng F. (2014). Vitamin B12-containing plant food sources for vegetarians. Nutrients.

[2] Hopp M.T., Schmalohr B.F., Kühl T., Detzel M.S., Wißbrock A., Imhof D. (2020). Heme Determination and Quantification Methods and Their Suitability for Practical Applications and Everyday Use. Anal. Chem..

[3] Caughey W.S., Ibers J.A. (1977). Crystal and Molecular Structure of the Free Base Porphyrin, Protoporphyrin IX Dimethyl Ester. J. Am. Chem. Soc..

[4] Calvano C.D., Ventura G., Cataldi T.R.I., Palmisano F. (2015). Improvement of chlorophyll identification in foodstuffs by MALDI ToF/ToF mass spectrometry using 1,5-diaminonaphthalene electron transfer secondary reaction matrix. Anal. Bioanal. Chem..

[5] Huang S.C., Hung C.F., Wu W.B., Chen B.H. (2008). Determination of chlorophylls and their derivatives in Gynostemma pentaphyllum Makino by liquid chromatography-mass spectrometry. J. Pharm. Biomed. Anal..

[6] Carlsson M.L.R., Kanagarajan S., Bülow L., Zhu L.H. (2020). Plant based production of myoglobin—A novel source of the muscle heme-protein. Sci. Rep..

[7] Xing Y., Gao S., Zhang X., Zang J. (2022). Dietary Heme-Containing Proteins: Structures, Applications, and Challenges. Foods.

[8] Shan L., Xu X., Zhang J., Cai P., Gao H., Lu Y., Shi J., Guo Y., Su Y. (2021). Increased hemoglobin and heme in MALDI-TOF MS analysis induce ferroptosis and promote degeneration of herniated human nucleus pulposus. Mol. Med..

[9] Hooda J., Shah A., Zhang L. (2014). Heme, an essential nutrient from dietary proteins, critically impacts diverse physiological and pathological processes. Nutrients.

[10] Gu S., Marianov A.N., Lu T., Zhong J. (2023). A review of the development of porphyrin-based catalysts for electrochemical CO_2_ reduction. Chem. Eng. J..

[11] Paolesse R., Nardis S., Monti D., Stefanelli M., Di Natale C. (2017). Porphyrinoids for Chemical Sensor Applications. Chem. Rev..

[12] Teo R.D., Hwang J.Y., Termini J., Gross Z., Gray H.B. (2017). Fighting Cancer with Corroles. Chem. Rev..

[13] Gregory P. (2000). Industrial applications of phthalocyanines. J. Porphyr. Phthalocyanines.

[14] Fernández C.C., Williams F.J. (2023). Reactions involving the central cavity of porphyrin molecules attached to self-assembled monolayers. Inorganica Chim. Acta.

[15] Zheng W., Shan N., Yu L., Wang X. (2008). UV–visible, fluorescence and EPR properties of porphyrins and metalloporphyrins. Dye Pigment..

[16] Wang J., Tan S., Liang Q., Guan H., Han Q., Ding M. (2019). Selective separation of bovine hemoglobin using magnetic mesoporous rare-earth silicate microspheres. Talanta.

[17] Fateen E., Abd-Elfattah A., Gouda A., Ragab L., Nazim W. (2011). Porphyrins profile by high performance liquid chromatography/electrospray ionization tandem mass spectrometry for the diagnosis of porphyria. Egypt. J. Med. Hum. Genet..

[18] Woltering M., Tulipani S., Boreham C.J., Walshe J., Schwark L., Grice K. (2016). Simultaneous quantitative analysis of Ni, VO, Cu, Zn and Mn geoporphyrins by liquid chromatography-high resolution multistage mass spectrometry: Method development and validation. Chem. Geol..

[19] Magi E., Ianni C., Rivaro P., Frache R. (2001). Determination of porphyrins and metalloporphyrins using liquid chromatography–diode array detection and mass spectrometry. J. Chromatogr. A.

[20] Monopoli A., Calvano C.D., Nacci A., Palmisano F. (2014). Boronic acid chemistry in MALDI MS: A step forward in designing a reactive matrix with molecular recognition capabilities. Chem. Commun..

[21] Bradshaw R., Bleay S., Clench M.R., Francese S. (2014). Direct detection of blood in fingermarks by MALDI MS profiling and Imaging. Sci. Justice.

[22] Srinivasan N., Haney C.A., Lindsey J.S., Zhang W., Chait B.T. (1999). Investigation of MALDI-TOF Mass Spectrometry of Diverse Synthetic Metalloporphyrins, Phthalocyanines and Multiporphyrin Arrays. J. Porphyr. Phthalocyanines.

[23] Kim T., Lee J., Kim J. (2015). Effect of laser intensity on the apparent isotope patterns of heme and peptide ions in MALDI-TOF MS. Int. J. Mass. Spectrom..

[24] Yang H.J., Park K.H., Shin S., Lee J.H., Park S., Kim H.S., Kim J. (2013). Characterization of heme ions using MALDI-TOF MS and MALDI FT-ICR MS. Int. J. Mass. Spectrom..

[25] Whiteaker J.R., Fenselau C.C., Fetterolf D., Steele D., Wilson D. (2004). Quantitative Determination of Heme for Forensic Characterization of Bacillus Spores Using Matrix-Assisted Laser Desorption/Ionization Time-of-Flight Mass Spectrometry. Anal. Chem..

[26] Yin Z., Sun B., Wang X., Cheng X., Hang W., Huang B. (2014). Comprehensive analysis of metalloporphyrins via high irradiance laser ionization time-of-flight mass spectrometry. J. Anal. At. Spectrom..

[27] Calvano C.D., Ventura G., Palmisano F., Cataldi T.R.I. (2016). 4-Chloro-α-cyanocinnamic acid is an efficient soft matrix for cyanocobalamin detection in foodstuffs by matrix-assisted laser desorption/ionization mass spectrometry (MALDI MS). J. Mass Spectrom..

[28] Calvano C.D., Ventura G., Trotta M., Bianco G., Cataldi T.R.I., Palmisano F. (2017). Electron-Transfer Secondary Reaction Matrices for MALDI MS Analysis of Bacteriochlorophyll a in Rhodobacter sphaeroides and Its Zinc and Copper Analogue Pigments. J. Am. Soc. Mass Spectrom..

[29] Calvano C.D., Capozzi M.A.M., Punzi A., Farinola G.M., Cataldi T.R.I., Palmisano F. (2018). 1,5-Diaminonaphtalene is a Highly Performing Electron-Transfer Secondary-Reaction Matrix for Laser Desorption Ionization Mass Spectrometry of Indolenine-Based Croconaines. ACS Omega.

[30] Calvano C.D., Monopoli A., Cataldi T.R.I., Palmisano F. (2018). MALDI matrices for low molecular weight compounds: An endless story? Anal. Bioanal. Chem..

[31] Buchler J.W., Smith K.M.E. (1975). Static coordination chemistry of metalloporphyrins. Porphyrins and Metalloporphyrins.

[32] Knochenmuss R., Porta Siegel T. (2021). An Introduction to MALDI Ionization Mechanisms for Users of Mass Spectrometry Imaging. MALDI Mass Spectrometry Imaging: From Fundamentals to Spatial Omics.

[33] Nazim Boutaghou M., Cole R.B. (2012). 9,10-Diphenylanthracene as a matrix for MALDI-MS electron transfer secondary reactions. J. Mass Spectrom..

[34] Wei J., Li H., Barrow M.P., O’Connor P.B. (2013). Structural characterization of chlorophyll-a by high resolution tandem mass spectrometry. J. Am. Soc. Mass Spectrom..

[35] Rigante E.C.L., Calvano C.D., Picca R.A., Modugno F., Cataldi T.R.I. (2023). An insight into spray paints for street art: Chemical characterization of two yellow varnishes by spectroscopic and MS-based spectrometric techniques. Vacuum.

[36] Zhang S., Chen Y., Liu J.A., Xiong S.X., Wang G.H., Chen J., Yang G.Q. (2009). New matrix of MALDI-TOF MS for analysis of small molecules. Chin. Chem. Lett..

[37] Copper Phthalocyanine, CI 74160. https://webbook.nist.gov/cgi/inchi?ID=C147148&Mask=200#Mass-Spec.

[38] Nickel Phthalocyanine. https://webbook.nist.gov/cgi/inchi?ID=C14055028&Mask=200.

[39] Achar B.N., Fohlen G.M., Lokesh K.S. (2003). Degradation study on the thermally stable nickel phthalocyanine sheet polymer. Polym. Degrad. Stab..

[40] Pashynska V.A., Van Den Heuvel H., Claeys M., Kosevich M.V. (2004). Characterization of noncovalent complexes of antimalarial agents of the artemisinin-type and FE(III)-Heme by electrospray mass spectrometry and collisional activation tandem mass spectrometry. J. Am. Soc. Mass Spectrom..

[41] Demirev P.A., Feldman A.B., Kongkasuriyachai D., Scholl P., Sullivan D., Kumar N. (2002). Detection of Malaria Parasites in Blood by Laser Desorption Mass Spectrometry. Anal. Chem..

[42] Li Y.T., Hsieh Y.L., Henion J.D., Ganem B. (1993). Studies on heme binding in myoglobin, hemoglobin, and cytochrome c by ion spray mass spectrometry. J. Am. Soc. Mass Spectrom..

[43] Chiavarino B., Crestoni M.E., Fornarini S., Rovira C. (2007). Protonated Heme. Chem.—A Eur. J..

[44] Shin S., Yang H.J., Kim J.H., Kim J., Lee J.H., Park K.H., Kim H.S., Kim J. (2012). Clarification of a peak at m/z 1634 from tryptically digested cytochrome c. J. Mass Spectrom..

[45] Zhang H., Yang F., Qian W.J., Brown R.N., Wang Y., Merkley E.D., Park J.H., Monroe M.E., Purvine S.O., Moore R.J. (2011). Identification of c-Type Heme-Containing Peptides Using Non-Activated Immobilized Metal Affinity Cchromatography Resin Enrichment and Higher-Energy Collisional Dissociation. Anal. Chem..

