# Supplementary material for: Matrix Selection Strategies for MALDI-TOF MS/MS Characterization of Cyclic Tetrapyrroles in Blood and Food Samples
Source: Molecules. 2024 Feb 15;29(4):868. doi: 10.3390/molecules29040868 (PMC10891649; doi:10.3390/molecules29040868)
Supplement: Supplementary file 1 [file molecules-29-00868-s001.zip › molecules-2826103-supplementary.pdf]

molecules

Supplementary Materials

Matrix Selection Strategies for MALDI-TOF MS/MS Characterization of Cyclic Tetrapyrroles in Blood and Food Samples

Mariachiara Bianco [1], Giovanni Ventura [1], Cosima Damiana Calvano [1,2,*], Ilario Losito [1,2], Tommaso R. I. Cataldi [1,2] and Antonio Monopoli [1,*]

[1] Department of Chemistry, University of Bari Aldo Moro, 70126 Bari, Italy; mariachiara.bianco@uniba.it (M.B.); giovanni.ventura@uniba.it (G.V.); ilario.losito@uniba.it (I.L.); tommaso.cataldi@uniba.it (T.R.I.C.)
[2] Interdepartmental Research Center (SMART), University of Bari Aldo Moro, 70126 Bari, Italy
* Correspondence: cosimadamiana.calvano@uniba.it (C.D.C.); antonio.monopoli@uniba.it (A.M.)

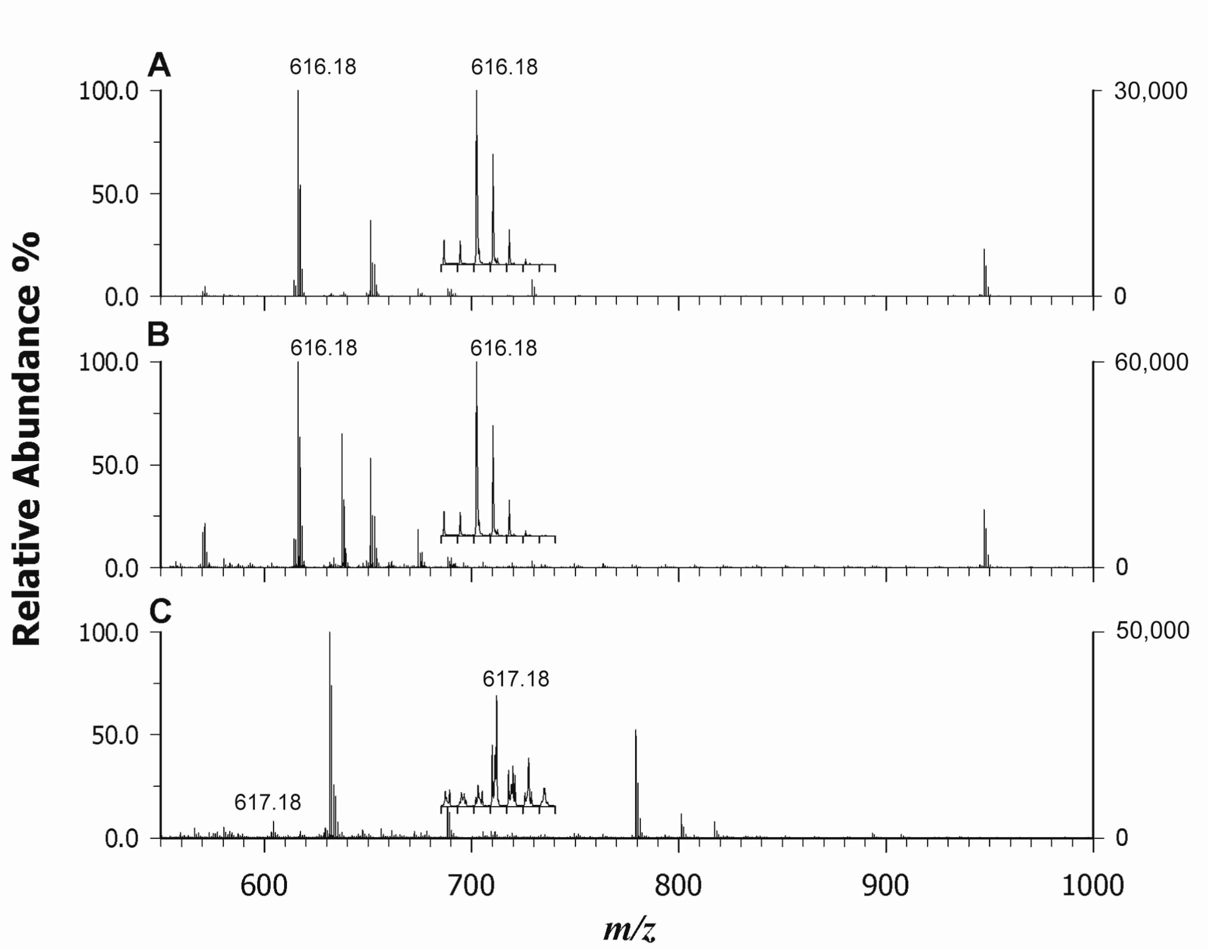

Figure S1. MALDI-ToF mass spectra in positive ion mode using 2-[(2E)-3-(4-tert-butylphenyl)-2-methylprop-2-enylidene]malononitrile (DCTB) as a matrix of heme b in hemoglobin (A), in myoglobin (B), and heme c in cytochrome C (C).

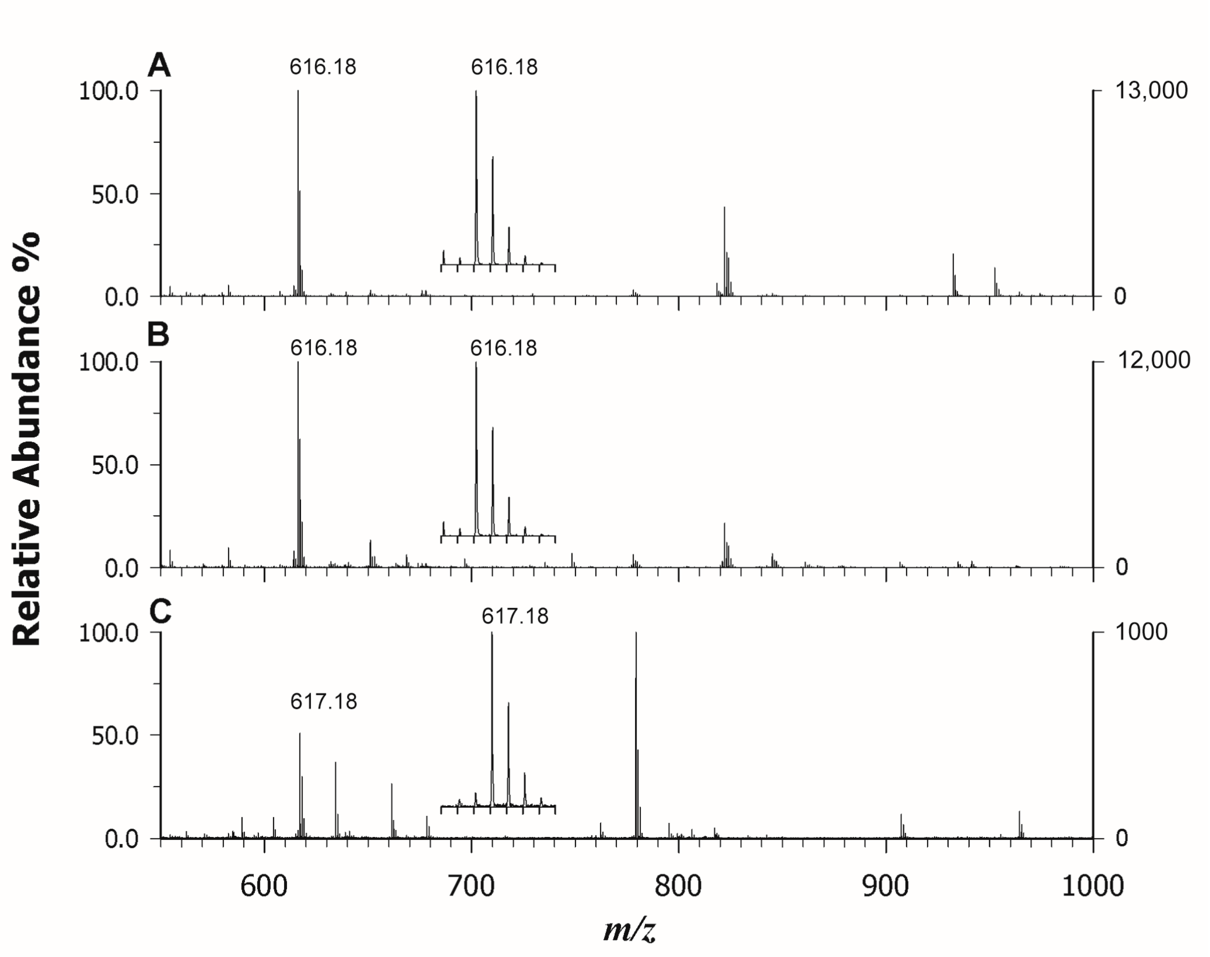

Figure S2. MALDI-ToF mass spectra in positive ion mode using CClCA as a matrix of heme b in hemoglobin (A), in myoglobin (B), and heme c in cytochrome C (C).

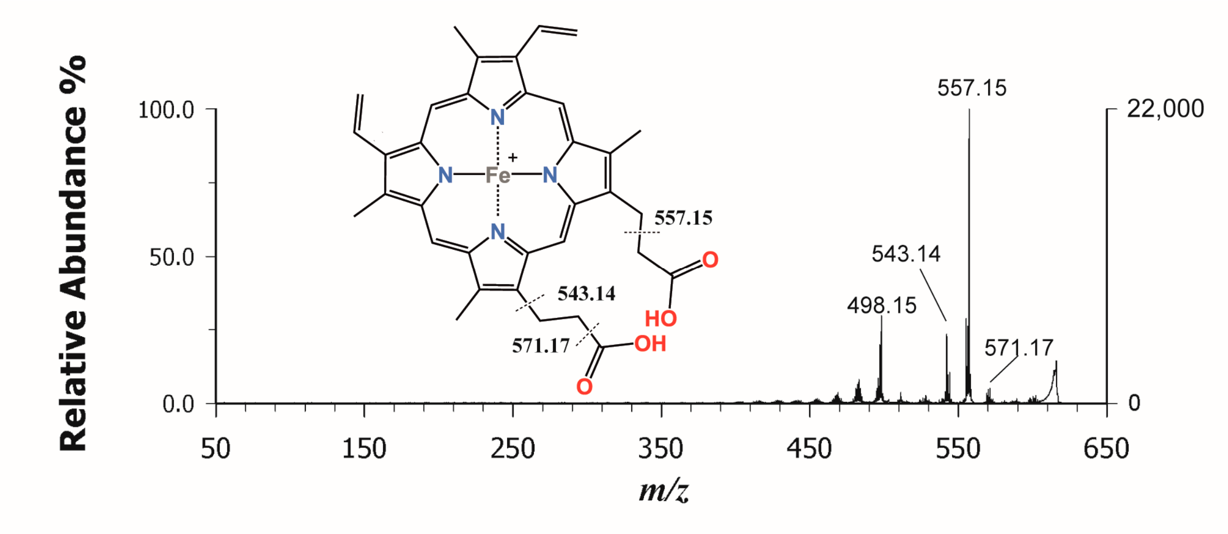

Figure S3. Tandem MS spectrum of heme b from hemoglobin standard protein at *m*/*z* 616.18 using CHCA as a matrix.
